# A Case of Isaacs Syndrome Developed After Thymectomy for Myasthenia Gravis

**DOI:** 10.7759/cureus.86696

**Published:** 2025-06-24

**Authors:** Hiroka Sakaguchi, Shunsuke Tsutsui, Kenta Aohara, Akiko Tamura, Yoshiaki Itoh

**Affiliations:** 1 Neurology, Osaka Metropolitan University, Osaka, JPN; 2 Neurology, Sumitomo Hospital, Osaka, JPN

**Keywords:** anti-caspr2 antibody, anti-lgi1 antibody, cold toe, myalgia, myokymia

## Abstract

A 54-year-old man was referred to our hospital due to calf pain and gait disturbance. Two years earlier, he had developed ptosis of the left eye. A routine health check conducted two years later revealed a thymoma with positive anti-acetylcholine receptor antibodies. He was diagnosed with myasthenia gravis and underwent thymectomy. Eight days after the surgery, he experienced involuntary calf muscle movements and pain, leading to worsening gait disturbance. Upon admission, he presented with cold toes, ptosis of the left eye, and continuous myokymia of both legs, which persisted during sleep. Laboratory tests revealed the presence of anti-contactin-associated protein 2 (anti-CASPR2) and anti-leucine-rich glioma-inactivated protein 1 (anti-LGI1) antibodies in the serum, along with myokymic discharges observed in electromyography. A diagnosis of Isaacs syndrome was made. The patient was treated with plasmapheresis and methylprednisolone pulse therapy, followed by intravenous immunoglobulin therapy, leading to the resolution of his symptoms. To our knowledge, this is the first reported case of Isaacs syndrome developing soon after thymectomy.

## Introduction

Isaacs syndrome, also known as acquired neuromyotonia, is an autoimmune disorder characterized by spontaneous and continuous muscle activity originating from peripheral nerves [1,2]. The first comprehensive description of the syndrome was provided by Isaacs in 1961 [3], who reported two cases of a previously unrecognized condition resembling myotonia. Electromyography performed under the influence of various test drugs strongly suggested that the discharges originated from peripheral nerves [3]. The autoimmune etiology of Isaacs syndrome was first reported by Sinha in 1991 [4]. More recently, autoantibodies against voltage-gated potassium channels (VGKCs) have been identified in 35-45% of patients with Isaacs syndrome [5,6]. VGKC antibodies were then identified in patients with Morvan syndrome, a rare disease characterized by neuromyotonia, dysautonomia, and encephalopathy with severe insomnia, as well as in those with limbic encephalitis [5]. Subsequently, it was shown that these antibodies were directed against three proteins bound tightly with VGKCs in detergent extracts of mammalian brain tissue: leucine-rich glioma-inactivated protein 1 (LGI1), contactin-associated protein 2 (CASPR2), and contactin [5]. Antibodies against LGI1 are strongly associated with limbic encephalitis, whereas antibodies against CASPR2, alone or occasionally with LGI1 antibodies, are frequently observed in patients with Morvan syndrome and Isaacs syndrome [5,7].

Herein, we report the first case of Isaacs syndrome with anti-LGI1 and anti-CASPR2 antibodies that developed after thymectomy for myasthenia gravis.

## Case presentation

A 54-year-old man was referred to our hospital with bilateral calf pain and gait disturbance. Two years earlier, he noticed ptosis of the left eye, which frequently appeared in the afternoon and worsened in the evening. He did not experience diplopia, speech disturbances, or fatigability in the extremities.

**Figure 1:**
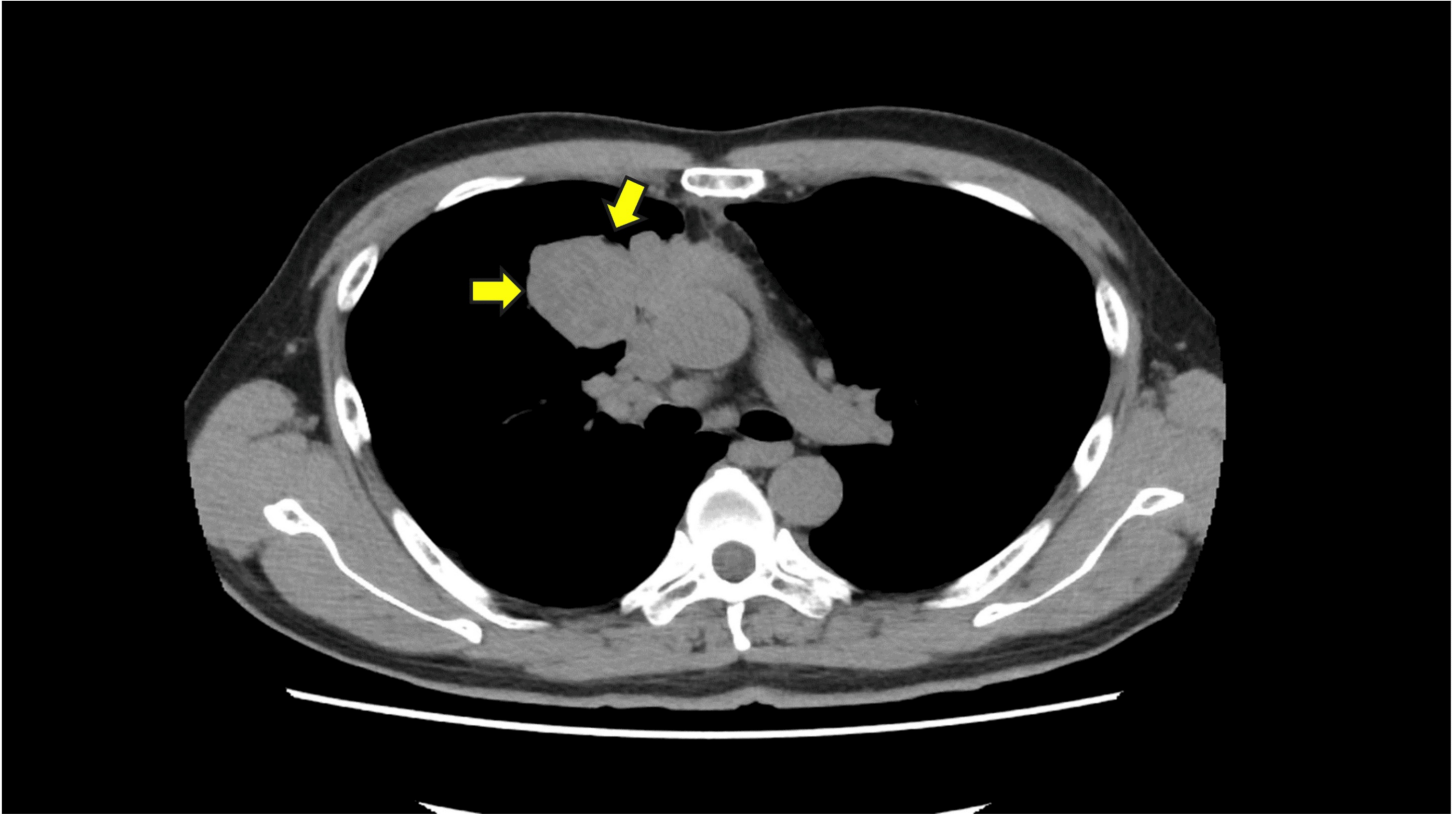
Thymoma on chest CT. Thymoma is found in the anterior mediastinum extending to the right lung (arrows).

Two years later, chest radiography performed during a routine health checkup revealed protrusion of the superior mediastinum to the right. Subsequent computed tomography and bronchoscopy confirmed a type B3 (T3N0M0) thymoma (Figure 1). Blood tests revealed an anti-acetylcholine receptor antibody level of 230 nmol/L (normal range <0.3 nmol/L). Based on these findings, he was diagnosed with myasthenia gravis associated with thymoma and underwent thymectomy without any precedent treatments for myasthenia gravis.

**Figure 2:**
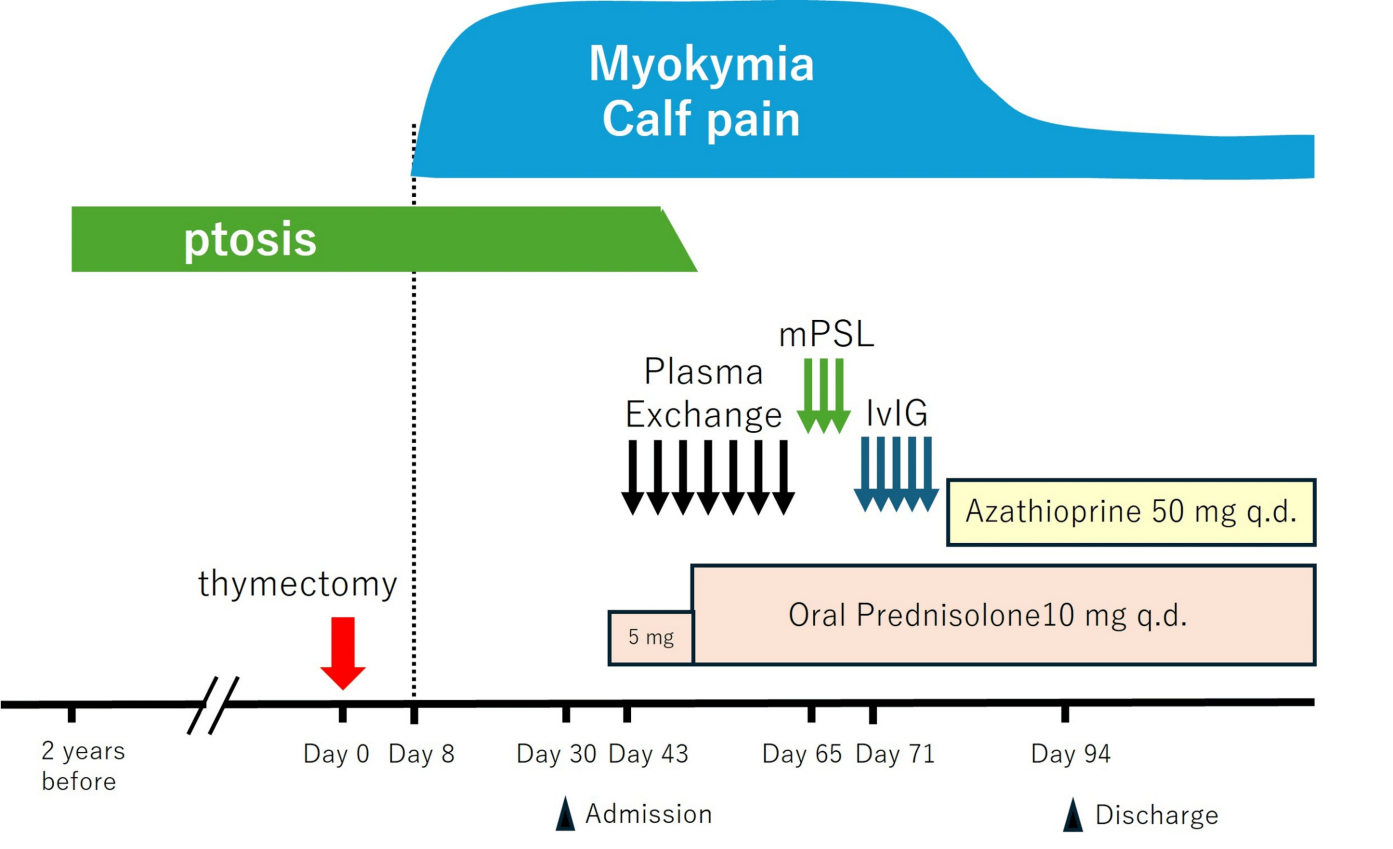
Clinical time course. Eight days after thymectomy (red arrow), the patient developed myokymia and calf pain. Plasma exchange was initiated on day 43, but myokymia remained unaffected even after the following methylprednisolone pulse therapy (mPSL), which began on day 65. Intravenous immunoglobulin (IvIG) treatment, started on day 71, finally relieved the symptoms. q.d.: every day.

Eight days after surgery, the patient began experiencing continuous involuntary twitching and pain in his calf muscles. The twitching persisted even during sleep and worsened with increasing physical activity. He also reported coldness in his toes, abnormal leg sensations, constipation, and insomnia. His muscle pain progressively worsened, leading to difficulty walking. He was admitted to our hospital 10 days after surgery (Figure 2).

On admission, the patient’s body temperature was 35.9℃. His blood pressure was 103/79 mmHg, and his pulse rate was regular at 78 beats per minute. No hyperhidrosis or skin discoloration was observed. No other abnormalities were noted on the physical examination.

**Figure 3:**
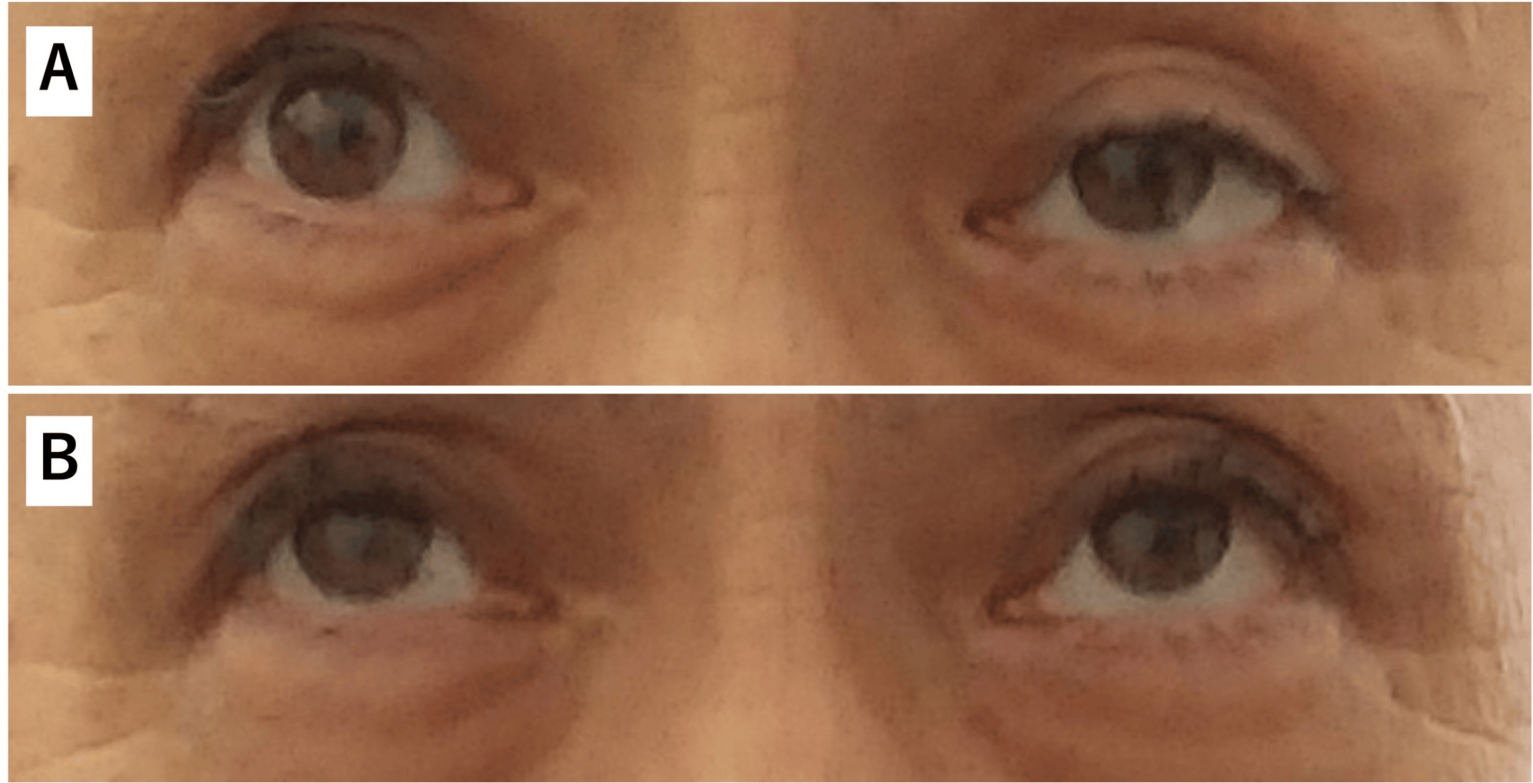
Edrophonium test. (A) Ptosis of the left eye remained unchanged following saline injection (control). (B) Ptosis resolved two minutes after edrophonium injection.

Neurological examination revealed that the patient was alert and oriented. Ptosis in the left eye was induced by an upward gaze (Figure 3A), whereas diplopia was rarely experienced following prolonged lateral gaze. Neither dysarthria nor dysphagia was present. Continuous myokymia was observed prominently in the lower legs and sparsely in the upper legs (Video 1). Neither percussion nor grip myotonia was observed. Muscle volume, strength, and tone were normal without evidence of fatigability. The patient did not exhibit ataxia in the extremities. Deep tendon reflexes were normal, and no pathological reflexes were observed. He reported coldness in his toes on both feet and paresthesia in his legs. He had difficulty standing up and walking because of leg pain.

**Video 1:** Myokymia in the right gastrocnemius muscle. Spontaneous twitching of a group of muscle fibers was randomly observed on the muscle surface at irregular intervals and in widespread muscles.

Most blood test results were within physiological range (Table 1). There was no evidence of an inflammatory response. The creatine kinase level was 122 U/L (59-248 U/L). Thyroid function was normal, although anti-thyroglobulin antibody was elevated at 12.6 IU/mL (normal <4.11 IU/mL). Serological testing for autoantibodies revealed positivity for anti-LGI1 and anti-CASPR2 antibodies. The antinuclear antibody level was 1:80 (normal <1:40) with a homogeneous pattern. Anti-glutamic acid decarboxylase antibody levels were <5.0 (normal <5.0 U/mL), and anti-acetylcholine receptor antibody levels were significantly elevated at 220 nmol/L (normal <0.3 nmol/L).

**Table 1:** Results of blood tests. LGI1: leucine-rich glioma-inactivated protein 1; CASPR2: contactin-associated protein 2.

Parameter	Measurement	Reference	Unit
WBC	8600	3300–8600	/μl
RBC	5.05	435–555	x 10^6^/μl
Hemoglobin	14.9	13.7–16.8	g/dL
Hematocrit	45.0	40.7–50.1	%
Platelet	331	15.8–34.8	x 10^3^/μl
D-dimer	2.5	0–1.0	μg/ml
C-reactive protein	0.07	0–0.14	mg/dL
Total protein	7.2	6.6–8.1	g/dL
Albumin	4.0	4.1–5.1	g/dL
Urea nitrogen	12	8–20	mg/dL
Creatinine	0.89	0.65–1.07	mg/dL
Sodium	134	138–145	mmol/L
Potassium	4.2	3.6–4.8	mmol/L
Chlorine	97	101–108	mmol/L
Calcium	10.6	8.8–10.1	mg/dL
Magnesium	2.0	1.8–2.4	mg/dL
Total bilirubin	0.9	0.4–1.5	mg/dL
Aspartate aminotransferase	29	13–30	U/L
Alanine aminotransferase	33	10–42	U/L
γ-glutamyl transferase	36	13–61	U/L
Lactate dehydrogenase	181	124–222	U/L
Creatine kinase	122	59–248	U/L
Blood sugar	107	73–109	mg/dL
Free T4	1.82	0.9–1.7	ng/dL
Thyroid-stimulating hormone	1.32	0.34–4.0	μIU/mL
Anti-thyroglobulin antibody	12.6	<4.11	IU/mL
Anti-thyroid peroxidase antibody	2.6	<16	IU/mL
Anti-LGI1 antibody	Present	Absent	
Anti-CASPR2 antibody	Present	Absent	
Antinuclear antibody	1:80	<1:40	
	Homogeneous		
Anti-glutamic acid decarboxylase antibody	<5.0	<5.0	U/mL
Anti-acetylcholine receptor antibody	220	<0.3	nmol/L

**Figure 4:**
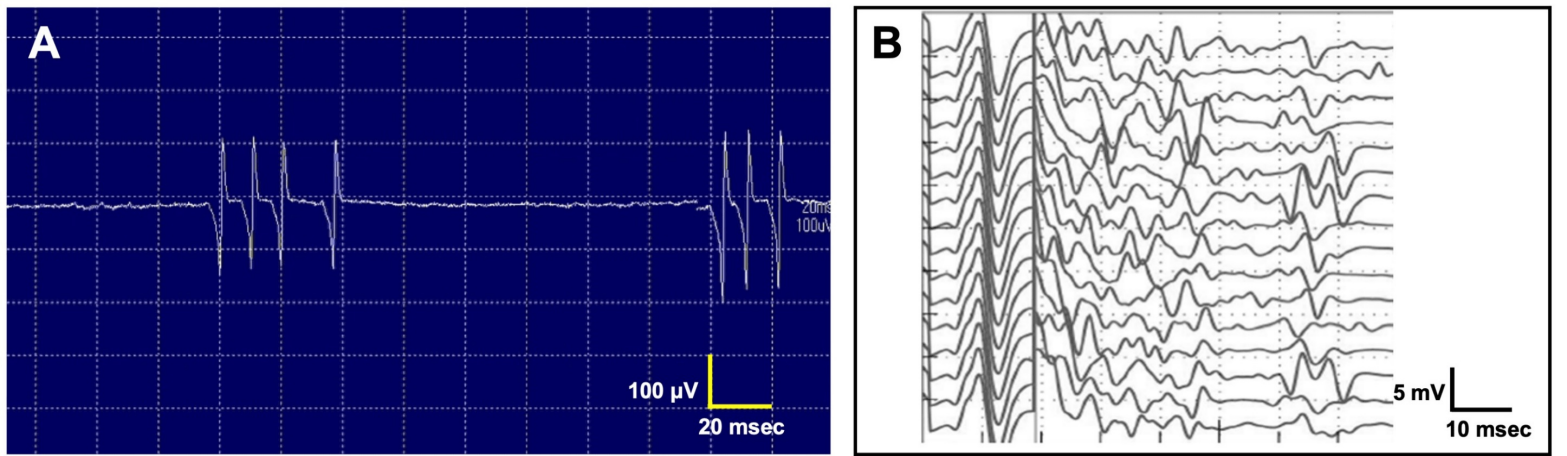
Electrophysiological studies. (A) Electromyography reveals multiple discharges in the gastrocnemius. The frequency is 50-100Hz. (B) F-wave recording shows frequent afterdischarges in the tibial nerve.

The edrophonium test, performed to evaluate ptosis, was positive (Figure 3B). Repetitive nerve stimulation showed a waning response in the facial nerve but not in the median, ulnar, radial, or accessory nerves. Electromyographic recordings revealed spontaneous, continuous, and irregularly occurring discharges of single motor units, appearing as doublets, triplets, or multiplets, firing at a high intraburst frequency of 30-300 Hz (Figure 4A). Characteristically, electrical nerve stimulation elicited increased spontaneous activity, observed as afterdischarges (Figure 4B).

In summary, a 54-year-old man developed calf myokymia after thymectomy. Both anti-CASPR2 and anti-LGI1 antibodies were positive. Electromyography revealed myokymic discharges. Based on these findings, the patient was diagnosed with Isaacs syndrome that developed shortly after thymectomy for myasthenia gravis.

Upon admission, the patient underwent seven sessions of plasma exchange at intervals of two to five days, accompanied by 5 mg of oral prednisolone once daily (Figure 2). The prednisolone dose was increased to 10 mg once daily during the third session of plasma exchange. Although the ptosis resolved quickly, myokymia and muscle pain persisted. He subsequently received three courses of steroid pulse therapy, each consisting of 1 g of intravenous methylprednisolone administered for three consecutive days. However, no improvement was observed in myokymia or muscle pain. Finally, the patient was treated with five courses of intravenous immunoglobulin (IVIG) therapy at 400 mg/kg/day, followed by oral azathioprine at 50 mg once daily. As the myokymia and calf pain gradually subsided, the patient was discharged home two months after admission.

## Discussion

The patient was diagnosed with Isaacs syndrome, which is characterized by prominent myokymia and muscle pain in the lower legs. The symptoms developed shortly after the surgical resection of the thymoma for myasthenia gravis. Anti-VGKC complex antibodies, including both anti-CASPR2 and anti-LGI1 antibodies, were positive. Electromyography revealed characteristic triplet and multiplet discharges, along with motor nerve hyperexcitability. The patient was successfully treated with IVIG.

Muscle twitching can be observed in various autoimmune and nonautoimmune conditions [2,8]. Among nonautoimmune diseases, lower motor neuron disorders, particularly those involving anterior horn cell damage, as seen in amyotrophic lateral sclerosis, often develop fasciculations [9]. Benign fasciculation syndrome is a harmless condition that is often triggered by stress, fatigue, or caffeine consumption [8]. Electrolyte imbalance, including low calcium, magnesium, and potassium levels, may also lead to muscle twitching [2]. Endocrine disorders such as hyperthyroidism, hypothyroidism, diabetes mellitus, and Addison’s disease may occasionally be associated with muscle twitching [10]. Exposure to toxins, including herbicides, insecticides, toluene, alcohol, and timber rattlesnake venom, can also induce muscle twitching [2]. Genetic conditions associated with muscle twitching include rippling muscle syndrome caused by caveolin-3 (*CAV3*) mutations [11] and episodic ataxia type 1 linked to mutations in the potassium channel gene *KCNA1* [12]. In the present case, most of these common differential diagnoses were excluded based on clinical history, neurological examination, and laboratory findings.

In contrast, Isaacs syndrome is considered an autoimmune disease. Associated conditions can be classified as paraneoplastic or non-paraneoplastic [2]. As in the present case, thymoma is the most frequent neoplastic disease, followed by small cell lung cancer, lymphoma, and plasmacytoma [2]. Non-paraneoplastic immune-mediated diseases associated with Isaacs syndrome include myasthenia gravis, Guillain-Barré syndrome, chronic inflammatory demyelinating polyneuropathy, Addison’s disease, hyperthyroidism, hypothyroidism, and systemic lupus erythematosus [2], among which myasthenia gravis preceded the Isaacs syndrome in the present case. Although the free T4 level was mildly elevated and the anti-thyroglobulin antibody was positive, thyroid disease was considered unlikely to be clinically relevant in this case, given the overall consistency of the laboratory data and clinical presentation.

Recently, autoantibodies against the VGKC complex have been reported in 35-45% of Isaacs syndrome cases [5,6]. The positivity rate for Isaacs syndrome associated with thymoma is reported to be as high as 80% based on immunoprecipitation assay [6]. Among the VGKC complex antibodies evaluated with cell-based assays, anti-CASPR2 alone, or both anti-CASPR2 and anti-LGI1 antibodies, as observed in the present case, are frequently associated with Isaacs syndrome [7]. In contrast, anti-LGI1 antibody alone is typically associated with limbic encephalitis [13].

The clinical features of double-positive (CASPR2 and LGI1) cases reported in the literature are summarized in Table 2 for comparison with the present case [7]. Age, sex, and most commonly reported clinical features were consistent with those observed in our patient. In contrast, thymoma and myasthenia gravis were reported in only 35% and 28% of cases, respectively (Table 2) [7]. In the present case, both conditions were identified before the development of Isaacs syndrome. Ptosis was the only symptom of myasthenia gravis that resolved quickly after thymectomy and plasma exchange. In contrast, Isaacs syndrome developed shortly after thymectomy. García-Merino et al. reported two cases of Isaacs syndrome with thymoma in which the symptoms remained unchanged or worsened after thymectomy [14]. Hart et al. described three patients who developed motor nerve hyperexcitability one to three years before being diagnosed with thymoma, whereas in seven patients, the symptoms appeared on average seven years after thymectomy (range: 1-22 years) [6]. To the best of our knowledge, no case has been reported in which motor nerve hyperexcitability developed soon after thymectomy, as in the present case. Outside the context of Isaacs syndrome, Sun et al. reported that six of 125 patients with thymoma without preoperative myasthenia gravis (4.8%) developed postoperative myasthenia gravis [15]. In four of these six patients, an increase in the anti-acetylcholine receptor antibody titer was observed after thymectomy, suggesting extrathymic activation of antibody production [15]. Similarly, in the present case, thymectomy might have triggered the extrathymic facilitation of anti-VGKC antibodies production from plasma cells, which might be controlled by the immune network of the bone marrow, spleen, and lymph nodes, although this mechanism could not be further confirmed by quantitative analysis of the anti-VGKC titer, which was not clinically available.

**Table 2:** Features of double-positive (CASPR2 and LGI1) patients reported in the literature as compared with the present case. * Demographic and clinical features of 46 patients described in the literature from 13 papers (Binks et al.) [7]. CASPR2: contactin-associated protein 2; LGI1: leucine-rich glioma-inactivated protein 1.

Category	Feature	Literature*	Present case
Demographics	Age (range)	46.7 (2-86)	54
	Male gender	28/37 (76%)	Male
Clinical presentation	Isaacs syndrome	27/37 (73%)	Present
	Morvan syndrome	20/37 (54%)	Absent
	Limbic encephalitis	3/38 (8%)	Absent
	Pain/paresthesia	21/34 (62%)	Present
	Peripheral neuropathy	12/31 (39%)	Absent
	Any dysautonomia	27/32 (73%)	Present
	Hyperhidrosis	22/30 (73%)	Absent
	Tachycardia/arrhythmia	9/16 (56%)	Absent
	Insomnia	20/28 (71%)	Present
	Seizures	8/39 (21%)	Absent
Accompanying tumor	Any tumor	16/35 (46%)	Present
	Thymoma	12/34 (35%)	Present
Autoimmune disease	Myasthenia gravis	7/25 (28%)	Present
Imaging	Normal brain MRI	16/17 (94%)	Present
Laboratory examination	Serum hyponatremia	8/29 (28%)	Absent

In a review of Isaacs syndrome, thymectomy was found to have minimal impact on the clinical severity of peripheral nerve hyperexcitability [2]. Plasma exchange is recommended as the first-line immunomodulatory treatment [1,16,17], followed by IVIG or methylprednisolone pulse therapy [18]. Oral prednisolone in combination with azathioprine may be an appropriate long-term maintenance therapy [1]. In the present case, thymectomy performed for myasthenia gravis in a patient without prior symptoms of Isaacs syndrome appeared to trigger the onset of Isaacs syndrome. IVIG following plasma exchange and methylprednisolone pulse therapy were effective in alleviating the symptoms, cautioning against thymectomy treatment for Isaacs syndrome.

## Conclusions

Herein, we present the first reported case of Isaacs syndrome that developed shortly after thymectomy for myasthenia gravis. A detailed evaluation revealed typical myokymic discharges on electromyography, motor nerve hyperexcitability on F-wave recordings, and double positivity for anti-CASPR2 and anti-LGI1 antibodies. Thymectomy may trigger extrathymic antibody production, leading to the development of Isaacs syndrome, as observed in some cases of myasthenia gravis. Anti-CASPR2 and anti-LGI1 antibodies should be evaluated in patients with Isaacs syndrome after thymectomy.
